# Abstract of the Proceedings of the Buffalo Medical Association

**Published:** 1855-11

**Authors:** Sanford B. Hunt


ART. II.—*Abstract of the Proceedings of the Buffalo Medical Association.*

TUESDAY EVENING, Oct. 2, 1855.

Association met.

Present—Drs. Strong, Newman, Rochester, White, Hamilton, Root, Wyckoff, Hawley, Lay, Ring, Hubbard, Wilcox, Hunt, Mixer, and Miner.

*Dr. Hamilton* presented a case of fractured clavicle:

I ask the attention of the society to the case of "bending of the clavicle," to which I made allusion at the last meeting. I remarked then, that it was not merely as a case of bending that I had thought it worthy of your notice, *but as a case in which the bone, after being bent and partially broken, had immediately resumed its form.* This is the peculiarity to which I wish also now to call your attention.

The child is present with his mother, and will submit to an examination. It is now eleven weeks since he fell down stairs, striking upon his right shoulder. The following day the mother noticed a swelling on the collar bone, and on the third day she took him to a physician, who applied a complete dressing, with axillary pad, sling, &c. On the fourth day she brought the child to me. The bandages, although they had been carefully and skillfully applied, as I believe, had already loosened and had been entirely removed by the mother.

I found a node-like projection on the clavicle at about the junction of the inner two-thirds with the outer third. It was three-quarters of an inch in length, hard, distinctly defined, and embraced the anterior and superior aspects of the bone: whether it extended around the entire circumference I could not determine positively, but I think not. The integuments above it were not discolored, no motion of the fragments was visible, nor was a motion perceptible to the touch, but only a very slight crepitus, which was, however, sufficiently distinct. The line of the bone was the same as in the opposite clavicle, and as if it had never been disturbed.

I applied no dressings, but have been permitted to see the child, from time to time, and these are the results:

On the ninth day crepitus had ceased. On the sixty-seventh day the node-like swelling (provisional callus) was nearly gone, and to-day, at the end of the eleventh week, you may still discover feeble traces of the callus, but the bone remains perfect in its form and length.

I have seen other similar cases, some of which you will find in the first part of the report on "Deformities after Fractures," made to the American Medical Association, including, also, a case reported to me by Caleb Green, Prof. of General Pathology, etc., at Geneva. I have under my care an infant in whom the same phenomena have occurred. The case before you only differs from most others in the fact that there was here present a slight crepitus. Only once or twice before do I remember to have found a crepitus where the bone has resumed its position spontaneously. This case is, therefore, the more valuable for our purpose, inasmuch as it is accompanied with conclusive evidence that the bone was bent, and in fact partially broken, while in other cases the evidences were less satisfactory.

It seems to establish the existence of a form of bending, or of partial fracture which I do not remember to have seen described: namely, a form in which the bone immediately resumes its original position by its own elasticity: and which will be recognized by a swelling or projection at the seat of fracture, seldom, if ever, discoverable immediately after the accident, and perhaps not very manifest until the second or third day; by the smooth, defined, hard, node-like character of the swelling; by its being limited, I think, generally to the anterior and superior parietes of the bone, or to those surfaces in the direction of which the fibres of the bone have given way; by the complete disappearance of this swelling in a few weeks, or months, and by the natural line of the bone remaining, from the first and throughout, unchanged.

NOTE.—In an article entitled "Fracture of the Skull in Children," published in this Journal, Dr. Hamilton has called especial attention to the spontaneous restoration of the *cranial* bones. See Buff. Med. Jour., vol 2, p. 347.

*Dr. Hubbard* reported a similar case in which no dressing was used, and which resulted very favorably.

*Dr. Wyckoff* reported a case to which he had been called that day. The patient, a child, fell from a high chair the day previous. No injury was discovered at the time. Dr. W., when called, found a slight protuberance (such as that in the case presented by Dr. Hamilton) at the point of fracture, near the middle of the bone. There was no displacement. He placed the arm in a sling, and confined it to the breast by a bandage around the body.

*Dr. Hamilton*, in answer to an inquiry from Dr. Hubbard, stated his general preference for Fox's apparatus—that he found it simple, and as effective as any other.

*Dr. Rochester* exhibited a pathological specimen of the larynx of a child, presented to him by Dr. Wm. Newell.

The history was imperfect. The child had been ill for a few days with sore throat and difficult respiration. When Dr. Newell was called he found it moribund, and it lived only four or five hours, during which time Dr. N. administered emetics with some stimulants.

The specimen, as presented, exhibited a narrow but long strip of false membrane floating in the larynx. It was not large, and was so loosely attached, that it would seem that the introduction of the probang would have detached and removed it.

The child, however, was so small that it could not have been induced to give the necessary consent, and, again, the opening of the rima-glottidis was so small that it was difficult to introduce even a catheter. The false membrane presented the usual microscopic appearances.

*Dr. White* reported a case of glossitis. A youth, aged 13 years, came a week since to his office with the tongue somewhat swollen, the muscles being pushed out laterally but not much elongated. In the hope of producing resolution, he prescribed a saline cathartic, and ordered tincture of iodine to be applied about the base of the tongue externally. He did not hear from him again until Friday, when he asked Dr. Hunt to accompany him on a visit. He found the tongue very much swollen and protruding from the mouth. Both sides were swollen, but the left side was soft, while the right was very dense and hard, it being evident that the inflammation was mostly on the right side.

The patient being by no means robust, the pulse being soft and not frequent, and no febrile reäction existing, it was decided to confine the treatment to a simple incision of the tongue, which was accordingly freely opened by a longitudinal incision a little external to the middle of the right side. It bled freely and was followed by much relief to the swelling, the patient being able to articulate much better. The decrease in swelling was most upon the external border of the tongue, and the incision was displaced by the diminution in size, so as to be quite upon the edge of the tongue.

On the succeeding day the tongue was found as badly swollen as before, with a circumscribed elevation upon the right of the median line, which excited the confident expectation of suppuration. This was disappointed. The incision was carried even more deeply than before through the hardened tissue, until the knife met with little resistance, but no pus was obtained. The incision, however, was followed by considerable relief, but the tongue was again swollen on the next day. He had not repeated the incision, and though the boy is now better, convalescence is not fully established. In the cases reported by Dr. T. D. Strong, of Westfield, who had had an unusual amount of experience in this very rare disease, the incisions had been followed by more permanent benefit.

*Dr. Miner* gave an account of an epidemic disease which prevailed two or three years since in the town of Athol, Mass., near which he was then residing. Some twenty or thirty children died with symptoms resembling croup, and other cases occurred in adjoining towns. There was, however, no formation of any false membrane. There was, uniformly, sore throat, and as the inflammation progressed downward, they proved rapidly fatal. In some cases the inflammatory action extended to the tongue, which became very much swollen, and in some cases protruded from the mouth. The progress of the disease was very rapid, many dying in from twelve to twenty-four hours. It was quite as fatal as membranous croup. It was not considered contagious, though some families had several cases. It was mostly confined to children, but he knew one instance in a young man of 18 years. *Post-mortem* examination revealed a highly inflamed condition of the trachea and bronchi.

*Dr. Wilcox* mentioned a case of glossitis to which he was called, but the patient refusing to have his tongue incised, it passed into other hands, and, as he understood, suppurated.

*Dr. White* related several cases as illustrative of the value of the microscope in the diagnosis of malignant or non-malignant disease of the uterus—a subject to which for a few years past he had devoted much attention. This means of diagnosis is usually more readily applied to uterine than to other disease.

Case I. Mrs. P., from Canada, had what had been called cancer of the uterus. The os was very much swollen, with extensive ulceration, accompanied by acute pain, sanguinolent and muco-purulent discharges, and the usual train of constitutional symptoms, sallow skin, debility, etc. She was just passing the turn of life.

The speculum and the general symptoms, indicated cancer, but relying on the negative evidence of the microscope, which exhibited no cancer cells, she was placed under treatment, remained here for two or three months, and recovered her health entirely.

Case II. Mrs. B., from Ohio, had suffered from uterine hæmorrhage for two and a-half years, and was rapidly sinking from loss of blood. She had an ulcer as large as a large almond upon the os, resembling cancer in its appearance. In this, as in the other case, the microscope afforded no evidence of cancer. She was under treatment but a few weeks, when the ulcer was entirely cured and cicatrized. There was still some leaking of blood from the cavity of the womb, but not one-tenth the previous quantity.

Two other cases from Genesee and Wyoming counties were mentioned, as similarly situated, and in which the negative evidence of the microscope was of great value as leading to a favorable prognosis.

In the treatment of these cases *potassa cum calce* was usually applied once or twice, the parts adjoining being guarded by a pledget of lint soaked in acetic acid, and carried up the speculum to neutralize any of the mixture which might flow down and do injury. An eschar was produced by this means, which, falling off, left the parts more disposed to heal. Nitras argenti was then usually applied, or tincture of iodine was used, or in cases where there was hæmorrhage the tincture ferri muriatis.

In the general treatment he made use of the iodides, tonics, the recumbent posture, and as early as it was admissible, exercise. A favorite prescription was

℞ Sulph. Ferri, ʒij.

Iod. Potassii., ʒiv.

Fl. Ext. Conii., ʒiv.

Syrup Zingiberis, ℥viij. M.

Dose one teaspoonful, three or four times daily.

As all the above cases had been called cancer, the value of the microscope was evident. Last Thursday (he would mention as showing the tolerance of the uterus) in attempting to check a hæmorrhage by carrying a stick of nitrate of silver into the uterus, he was so unfortunate as to break it, and leave a piece weighing as much as 10 grs. in the cavity. No disagreeable symptoms followed.

*Dr. Hamilton* reported a case of staining of the conjunctiva from long continued use of the nitrate of silver, which had been applied to the eyes for a period of about two years.

*Dr. Lay* had seen a similar case.

*Dr. Wyckoff* mentioned a case of obstinate retention of urine in an unmarried servant girl. She had been in perfect health until she had had intermittent fever, having also taken cold during her menstrual period. The retention was very obstinate, and required the catheter, which needed to be introduced with some force, owing to resistance. The catheter is now introduced twice a day. Dr. W. can assign no cause for the retention.

*Dr. White* recommended cantharides.

*Dr. Mixer* had seen a case attended by complete paralysis of both extremities. It terminated fatally.

*Dr. Rochester* proposed Dr. J. N. Brown for membership.

*Dr. Newman* moved that "when we adjourn, we adjourn to meet two weeks from this evening." Lost.

And the association then adjourned.

SANFORD B. HUNT, Secretary.

